# Role of innate signalling pathways in the immunogenicity of alphaviral replicon-based vaccines

**DOI:** 10.1186/1743-422X-8-36

**Published:** 2011-01-24

**Authors:** Tanja I Näslund, Linda Kostic, Eva KL Nordström, Margaret Chen, Peter Liljeström

**Affiliations:** 1 Department of Microbiology, Tumor and Cell Biology, Karolinska Institutet, Nobels väg 16, 171 77 Stockholm, Sweden; 2 Swedish Institute for Infectious Disease Control, Sweden; 3 BioArctic Neuroscience AB, Warfvingesväg 39, 112 51 Stockholm, Sweden; 4 Department of Dental Medicine, Karolinska Institutet, Sweden

## Abstract

**Background:**

Alphaviral replicon-based vectors induce potent immune responses both when given as viral particles (VREP) or as DNA (DREP). It has been suggested that the strong immune stimulatory effect induced by these types of vectors is mediated by induction of danger signals and activation of innate signalling pathways due to the replicase activity. To investigate the innate signalling pathways involved, mice deficient in either toll-like receptors or downstream innate signalling molecules were immunized with DREP or VREP.

**Results:**

We show that the induction of a CD8^+ ^T cell response did not require functional TLR3 or MyD88 signalling. However, IRF3, converging several innate signalling pathways and important for generation of pro-inflammatory cytokines and type I IFNs, was needed for obtaining a robust primary immune response. Interestingly, type I interferon (IFN), induced by most innate signalling pathways, had a suppressing effect on both the primary and memory T cell responses after DREP and VREP immunization.

**Conclusions:**

We show that alphaviral replicon-based vectors activate multiple innate signalling pathways, which both activate and restrict the induced immune response. These results further show that there is a delicate balance in the strength of innate signalling and induction of adaptive immune responses that should be taken into consideration when innate signalling molecules, such as type I IFNs, are used as vaccine adjuvant.

## Introduction

Alphaviral replicon-based vectors are attractive vaccine candidates since they induce strong immune responses in various animal models. The alphaviral replicon encodes an alphavirus replicase, an RNA polymerase, which strongly amplifies the replicon encoded transgene RNA resulting in high heterologous antigen production. Initially, the superior immune response induced by these types of vectors was attributed to abundant antigen production [1,2]. However, the replicase activity also leads to the formation of double stranded RNA (dsRNA), which induces activation and cross-priming of viral associated antigens in CD8α^+ ^dendritic cells (DCs) [3]. Hence, it is now becoming increasingly clear that the immunogenicity of alphaviral replicon-based vectors is due to activation of innate immune responses, rather than increased antigen production.

We have previously used alphaviral replicon-based vaccines administered as viral particles capable of one round of replication (VREPs) [4]. These VREPs, based on Semliki Forest virus (SFV), induce strong antibody and cellular responses in animals [5-10]. SFV, being a RNA virus, may target several innate signalling pathways [11,12] including toll-like receptor (TLR) 3 and 7, as well as cytoplasmic receptors of the RIG-I-like receptor (RLR) family [13]. We have shown that replication of VREP generates double-stranded (ds) RNA intermediates, and that immunization of mice with VREP infected Vero cells activates the TLR3 pathway leading to enhanced cross-priming [3]. However, immune activation was only partly dependent on TLR3, suggesting that other innate signalling pathways are involved. Other studies with RNA viruses have suggested that the MyD88 and TLR3 pathways are targeted [14-16]. However, recent results indicate that MyD88 and TLR3 pathways may be dispensable for generation of T cell responses after VREP immunization [17]. Thus, it is not clear which innate signalling pathways that are activated after VREP immunization. Engagement of TLRs and RLRs results in production of type I interferon (IFN) and pro-inflammatory cytokines. The release of type I IFN has been shown to amplify the innate immune responses and to be a potent inducer of the adaptive immune response by activation of DCs, T- and B-cells [18-21], and has been suggested to be a crucial signalling molecule for the generation of a potent immune response. We have shown that immunization with VREP also induces type I IFNs [22]. However, the impact of type I IFNs on alphaviral replicon-based immunogens is not clear.

Use of naked DNA for vaccination has gained much attention, in particular following early promising pre-clinical results in mice. However, later experiments performed with non-human primates and the first human clinical trials gave rather disappointing results [23-25]. Conventional DNA (convDNA) vaccines do target a number of innate signalling receptors, including toll-like receptors (TLR9) and various cytoplasmic receptors (e.g. DAI and AIM2) [26,27], but it may be that at conventional delivery doses, these signals are not strong enough to induce a robust immune response. Inclusion of elements resulting in apoptosis [28-30] or expression of interferon regulatory factors [31], events normally occurring during viral infections, has been shown to increase the immunogenicity of convDNAs, and suggests that one possible way to improve convDNA vaccines would be by mimicking a virus infection.

In our previous studies we have demonstrated that the immunogenicity of naked DNA is significantly improved by inclusion of the alphaviral replicon into convDNA vectors, constructing an alphavirus replicon-based DNA (DREP) vector [32-37]. This suggests that the viral replicase activity could contribute to the enhanced immunogenicity of the replicon-based vectors. While immunization with VREP particles do target various innate pathways it has not yet been investigated in head-to-head comparisons if these are the same for DREP vaccines. In this study we investigated if innate signalling pathways are important for the immunogenicity of alphaviral replicon-based vector immunization.

In this study we show that many of the innate receptors, at least on their own, are dispensable for induction of CD8^+ ^T cell responses both after DREP and VREP immunization. In contrast, IRF3, an important signalling molecule for the induction of type I IFNs and pro-inflammatory cytokines, is needed for a fullminant immune response. Interestingly, the immune responses are suppressed in both DREP and VREP immunized mice by type I IFNs. In conclusion, the immune response is affected by the TLR and RLR downstream signalling molecule IRF3 and type I IFNs, suggesting that multiple innate receptors are involved after replicon-based vaccine administration.

## Materials and methods

### Mice and immunizations

MyD88 knock-out (KO) [38], IRF3 KO [39] and corresponding wild type mice, C57Bl/6, and IFN-AR1 KO [40] mice and corresponding wild type mice, Sv129, were bred and kept at the animal house at Karolinska Institutet, Sweden. The TLR3 KO mice [41] and corresponding wild type mice, C57Bl/6:Sv129, were bred and kept at the animal house at the Swedish Institute for Infectious Disease Control, Sweden. Female mice, 6-12 weeks old were immunized intramuscularly (i.m.) under pathogen-free conditions with DREP-OVA (1, 10 or 50 μg), deltaREP-OVA (50 μg) or VREP-OVA (10^6 ^infectious units (IU)) in a total volume of 100 μl divided equally between both hind legs. The DREP-OVA and deltaREP-OVA DNA were diluted in sterile physiological 0.9% sodium chloride solution and the VREP-OVA viral particles in sterile PBS (Gibco, Invitrogen, Carlsbad, California). Animal care and treatment were in accordance with standards approved by the local ethics committee (Stockholms norra djurförsöksetiska nämnd).

### DNA and viruses

The DREP-OVA construct was made by cloning the *ova *encoding gene, coding for a cytoplasmic non-secreted form of OVA protein lacking the signal peptide, by *BglII *and *NotI *restriction digestion and T4 DNA ligase reaction. The deltaREP-OVA construct was made from the DREP-OVA construct by deletion of the region corresponding to the CMV promotor and the SFV replicase by *BamHI *restriction digestion, Klenow fill-in reaction and *AseI *restriction digestion. The CMV promoter from the pBK-LacZ plasmid was inserted into the deltaREP-OVA construct by *NheI *restriction digestion and Klenow fill-in reaction and *AseI *restriction digestion. All enzymes were obtained from NE Biolabs, Ipswich, MA. Plasmids were purified with Endotoxin free Mega-prep kit (Qiagen, Hilden, Germany) and preparations with endotoxin levels <0.1EU/μg DNA were used for immunization. The SFV two-helper RNA system has been described previously [42].

### ELISpot

IFN-γ ELISpot analysis was performed on freshly isolated splenocytes as described previously [10]. Splenocyte single-cell suspensions were treated with Red Blood Cell lysing buffer and re-suspended in RPMI media supplemented with 2 mM L-glutamine, 2 mM Penicillin-Streptomycin (all from Sigma-Aldrich, St. Louis, MO) and 10% FCS (Gibco, Invitrogen, Carlsbad, California) (complete media). Splenocytes (2 × 10^5^) from individual mice were added to Multiscreen IP plates (Millipore, Billerica, MA) coated with anti-mouse-IFN-γ monoclonal antibody (AN18) (Mabtech AB, Nacka strand, Sweden) and stimulated with media or 2 μg/ml OVA peptide (SIINFEKL) (Proimmune, Oxford, UK) or 2 μg/ml Concanavalin A (Sigma-Aldrich, St. Louis, MO) for 20 hours. The plates were thereafter developed with biotinylated anti-mouse-IFN-γ monoclonal antibody (R4-6A2) (Mabtech AB, Nacka strand, Sweden), Vectastain Elite ABC kit (Immunkemi F&D AB, Järfälla, Sweden) and AEC substrate (Sigma-Aldrich, St. Louis, MO). The spots were counted using an ELISpot reader (Axioplan 2 Imaging; Zeiss) and expressed as spot forming cells (SFC) per 10^6 ^splenocytes. A value equal to or greater than 55 spots per million splenocytes in the peptide wells is regarded a positive IFNγ response. Mice with media responses higher than 50 spots in the IFNγ ELISpot were omitted from further analysis.

### Statistics

Statistical analysis was performed using the GraphPad Prism 5 software (GraphPad Software Inc., La Jolla, CA). To test for statistical significance nonparametric two-tailed Mann-Whitney analysis was performed.

## Results

### Induction of CD8^+ ^T cell responses after deltaREP and DREP immunization

**Figure 1:**
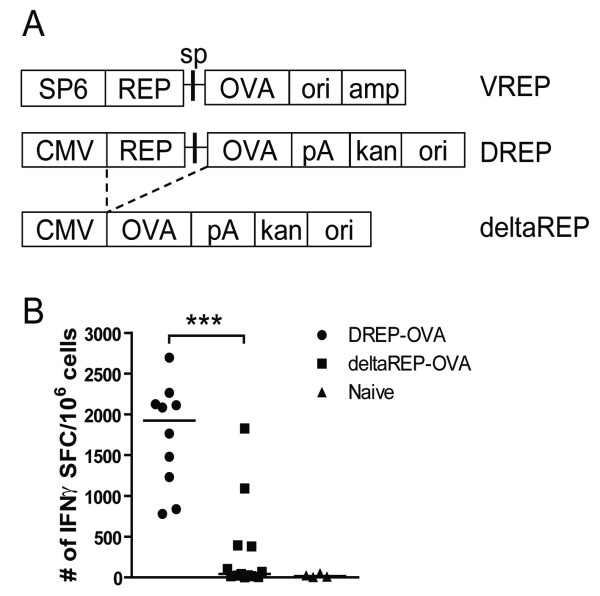
**Schematic representation of constructs and CD8**^**+ **^**T cell responses induced after deltaREP-OVA and DREP-OVA immunization**. (A) Illustration of VREP-OVA, DREP-OVA and deltaREP-OVA constructs. SP6 = SP6 RNA-polymerase promotor, CMV = cytomegalovirus promotor, REP = SFV replicase, sp = SFV subgenomic promoter, OVA = ovalbumine gene, ori = pUC origin, amp = ampicillin, pA = SV40 late polyadenylation signal, kan = kanamycin. Dotted lines denote deletion of REP from DREP-OVA to generate deltaREP-OVA. (B) OVA-specific CD8^+ ^IFNγ T cell responses in freshly isolated splenocytes 10 days after immunization with DREP-OVA (black circles (●)), deltaREP-OVA (black squares (■)) or naïve (black triangles (▲)) C57Bl/6 mice, measured by ELISpot. Values are expressed as numbers of IFNγ spot forming cells (SFC) per million splenocytes. Each symbol represents an individual mouse and the group median values are indicated by bars. Data were pooled from two experiments with 5 to 8 mice per group (B). The statistical difference between the groups were p < 0.001 (***).

We have previously demonstrated that SFV-based DREPs are more immunogenic in comparison to convDNA vectors. However, the different backbone compositions between the vectors were not considered in those studies [32,33]. To create a convDNA-like vaccine vector to be compared with DREP, a deltaREP vector was constructed by deleting the replicase region from DREP (Figure 1A). While the vectors certainly have a significant size difference, this strategy was chosen as a best effort to be able to compare vectors with (DREP) or without (deltaREP) replicase activity, sharing the same backbone. To be able to compare the immunogenicity of DREP vs deltaREP, the *ova *gene was inserted into both vectors (Figure 1A). In DREP the full-length RNA replicon is expressed from the CMV promoter, whereas the OVA protein is expressed by the viral replicase from the subgenomic promoter. In contrast, in the deltaREP construct the OVA protein is expressed directly under the CMV promoter. When transfected into BHK cells at similar mass (μg), both plasmids expressed the OVA antigen in similar amounts per cell (data not shown).

To compare the immunogenicity of the two DNA vaccines, C57Bl/6 mice were immunized with 50 μg of DNA and the splenic OVA-specific CD8^+ ^T cell responses were measured by IFNγ ELISpot 10 days post immunization. After deltaREP-OVA immunization, only a few mice responded to the Kb-restricted OVA SIINFEKL peptide. In contrast, all mice responded to DREP-OVA immunization, with significantly higher numbers of IFNγ producing CD8^+ ^T cells (p < 0.001) (Figure 1B). This result confirms previous studies that DREP is indeed more immunogenic than deltaREP (convDNA vaccines) [32,33].

### Replicon induced CD8^+ ^T cell responses in the absence of TLR signalling

**Figure 2:**
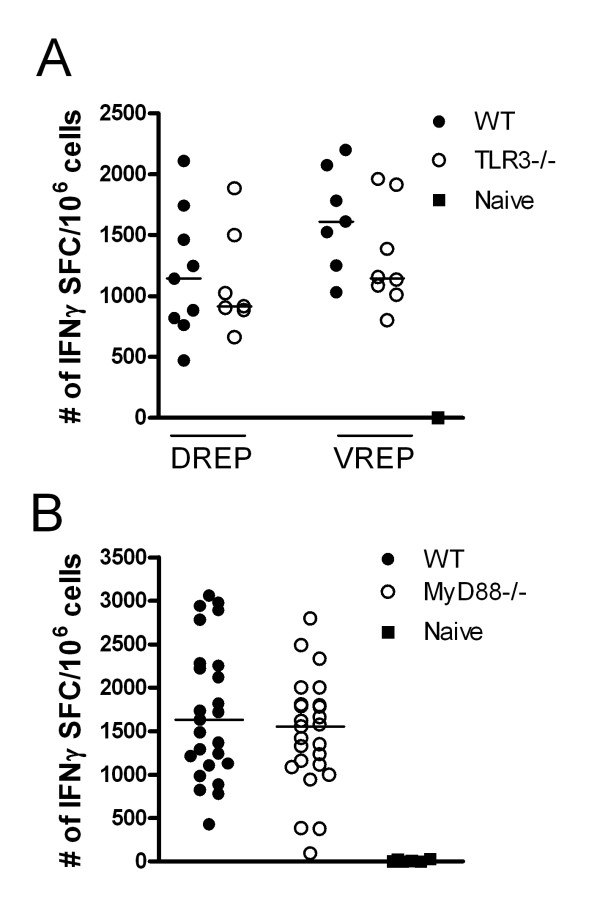
**OVA-specific CD8**^**+ **^**T cells in spleen 10 days after replicon immunization in wild type, TLR3KO and MyD88KO mice**. The CD8^+ ^T cell responses were measured in wild type, TLR3 KO (A) and MyD88 KO (B) mice. The numbers of OVA-specific CD8^+ ^T cells were measured by IFNγ ELISpot in wild type (black circles (●)), KO (open circles (○)) and naïve (black squares (■)) mice. Data were pooled from two experiments with 3 to 5 mice per group (A) and from three experiments with 5 to 10 mice per group (B). Values are expressed as numbers of IFNγ SFC per million splenocytes. Each symbol represents an individual mouse and the group median values are indicated by bars. No statistical difference was detected between the groups of mice.

Earlier studies investigating innate signalling pathways by replicon vectors have used VREP particles, whereas it has not been investigated in parallel if the same innate signalling pathways are activated by DREP. In order to investigate the involvement of toll-like receptor (TLR) and RIG-I-like receptor (RLR) family signalling in DREP and VREP induced immunity, we used TLR knock-out (KO) mice or mice lacking innate signalling molecules presumingly activated by VREP and DREP vectors. As alphaviral replicon-based vectors are known to generate dsRNA intermediates that could serve as TLR3 ligands [3,43], we first analyzed immune responses in wild type mice and TLR3 KO mice immunized with DREP-OVA or VREP-OVA. The OVA-specific CD8^+ ^T cell IFNγ response was measured in the spleen 10 days post immunization. No statistical significant difference was detected between the wild type and KO groups of mice after DREP-OVA or VREP-OVA immunization, although there was a tendency towards lower responses in the TLR3 KO groups (Figure 2A).

Another possibility is that TLR7 signalling could be involved, since DREP produces single-stranded RNA in the transfected cell. We therefore employed MyD88 KO mice which abolish signalling through TLR7. Again, we found no differences between wild type and MyD88 KO DREP-OVA immunized mice, suggesting that MyD88 dependent pathways, such as the TLR7 signalling pathway, are not crucial for generation of a strong CD8^+ ^T cell response (Figure 2B). Yet another TLR that is signalling via MyD88 and might be targeted by DREP, since DREP is administered as naked DNA, is TLR9. However, no significant difference was found between wild type and TLR9 deficient mice (data not shown).

Taken together, TLR3 and MyD88-dependent receptors are not crucial for the induction of a CD8^+ ^T cell response after VREP and DREP immunization.

### Replicon induced CD8^+ ^T cell responses in the absence of IRF3 signalling

**Figure 3:**
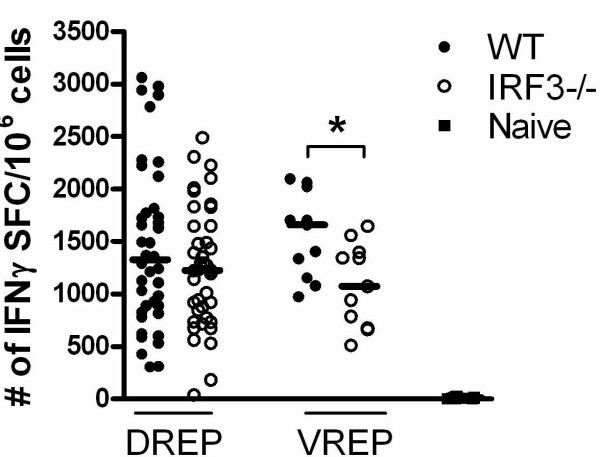
**OVA-specific splenic CD8**^**+ **^**T cell responses 10 days after replicon immunization in wild type and IRF3KO mice**. The CD8^+ ^T cell responses were measured in wild type (black circles (●)), IRF3 KO (open circles (○)) and naïve (black squares (■)) mice, 10 days post-immunization. The numbers of OVA-specific CD8^+ ^T cells were investigated by IFNγ ELISpot. Data are pooled from four experiments, with 5 to 10 mice per group. Values are expressed as numbers of IFNγ SFC per million splenocytes. Each symbol represents an individual mouse and the group median values are indicated by bars. The statistical difference between the groups were p < 0.05 (*).

RNA produced in viral infected cells not only targets the TLR3 pathway, but also signals through the RIG-I-like receptor (RLR) family, resulting in the induction of type I IFNs and pro-inflammatory cytokines via IRF3. It has recently been shown that VREP is recognised by the RLRs, MDA5 and RIG-I [44]. We have recently shown that lack of IRF3 results in reduced type I IFN levels and delayed type I IFN synthesis by VREP in DCs *in vitro *[22]. To investigate the importance of IRF3 *in vivo *we immunized wild type and IRF3 KO mice with DREP-OVA or VREP-OVA, and the OVA-specific CD8^+ ^T cell responses were measured 10 days post-immunization. There was a tendency towards lower responses in the IRF3 KO mice compared to wild type mice after DREP-OVA immunization, although there was no statistical significant difference (Figure 3). In contrast, VREP-OVA immunization induced statistically significantly lower level of IFNγ producing OVA-specific CD8^+ ^T cells in IRF3 KO mice compared to wild type mice (p < 0.05) (Figure 3), indicating that lack of type I IFN and pro-inflammatory cytokines reduce the level of the immune response.

### Replicon induced CD8^+ ^T cell responses in the absence of the type I interferon receptor

**Figure 4:**
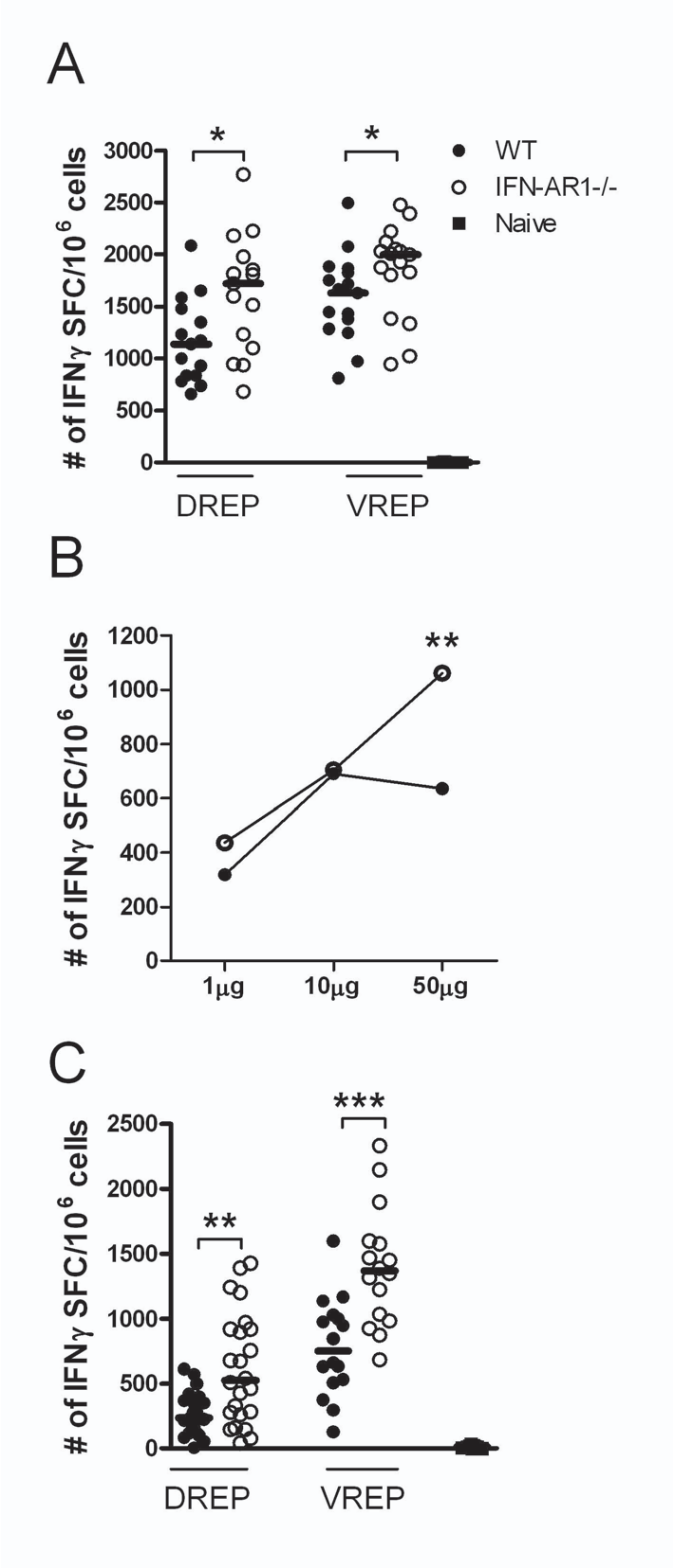
**OVA-specific CD8**^**+ **^**T cells in spleen after replicon immunization at primary peak and memory responses in wild type and IFN-AR1 KO mice**. The CD8^+ ^T cell response was measured 10 days (A) and (B) and five weeks post-immunization (C), in wild type (black circles (●)), IFN-AR1 KO (open circles (○)) and naïve (black squares (■)) mice. The numbers of OVA-specific CD8^+ ^T cells were measured by IFNγ ELISpot (A-C). In (A) and (C) mice were immunized with 50 μg DREP-OVA and in (B) with 1, 10 or 50 μg DREP-OVA. Each symbol represents an individual mouse and the group median values are indicated by bars in (A) and (C). In (B) the group median values are indicated by circles. Values are expressed as numbers of IFNγ SFC per million splenocytes. Data are pooled from four experiments, with 5 to 10 mice per group (A) and two experiments with 10 mice per group (B), and three experiments with 5 to 10 mice per group (C). The statistical difference between the groups were p < 0.05 (*), p < 0.01 (**) and p < 0.001 (***).

Type I IFNs have been shown to be potent inducers of both innate and adaptive immune responses [18-21] and we have previously shown that VREP strongly induces type I IFNs *in vivo *[22]. Type I IFNs have certainly been considered as vaccine adjuvants [45] and IFN stimulatory elements (IRF3, IRF7, TLR9) combined in/with convDNA vaccines have resulted in significantly increased T cell responses [31,46,47]. Since mice lacking IRF3 did show a reduced capacity to induce a CD8^+ ^T cell response and IRF3 is crucial for the generation of type I IFNs, we wanted to investigate whether IFN type I is important for the immune effect by VREP and DREP. Moreover, the effect of several TLRs (TLR3, 7 and 9) and RLRs converge into a type I IFN response. Therefore, we utilized mice lacking a functional IFNα/β receptor (IFN-AR1 KO mice), rendering these mice unresponsive to type I IFNs. Wild type and IFN-AR1 KO mice were immunized with DREP-OVA or VREP-OVA and the OVA-specific CD8^+ ^T cell responses were analyzed 10 days post-immunization. Surprisingly, the IFN-AR1 KO mice showed significantly higher T cell responses in comparison to wild type mice, both after DREP-OVA (p < 0.05) and VREP-OVA (p < 0.05) immunization (Figure 4A), indicating that type I IFNs suppress the immune response. To investigate if there was a lower dose limit where DREP-OVA would not induce suppressive amounts of type I IFNs, wild type and IFN-AR1 KO mice were immunized with lower doses of DREP-OVA (1 μg and 10 μg in addition to 50 μg, used elsewhere in the study). From these experiments it became clear that type I IFN did have a suppressive effect at higher DNA doses, as the numbers of OVA-specific CD8^+ ^IFNγ producing T cells in the wild type mice reached a plateau at doses exceeding 10 μg DREP-OVA (Figure 4B). In contrast, the CD8^+ ^T cell response increased with escalating doses of DREP-OVA in IFN-AR1 KO mice, resulting in significantly higher level of IFNγ producing CD8^+ ^T cells in IFN-AR1 KO mice compared to wild type mice at the dose of 50 μg DREP-OVA (p < 0.01).

Since lack of the type I IFN receptor had a pronounced effect on the primary T cell response and several reports have shown that type I IFNs are important for the maintenance of memory cells [48,49], we next investigated whether lack of the type I IFN receptor had any effect on the memory pool. Wild type and IFN-AR1 KO mice were immunized with DREP-OVA or VREP-OVA and five weeks post immunization, the splenic memory response was analysed by IFNγ ELISpot (Figure 4C). As was the case in the primary response, statistically significantly higher numbers of OVA-specific IFNγ producing CD8^+ ^T cells were detected in IFN-AR1 KO mice in comparison to wild type mice, both after DREP-OVA (p < 0.01) and VREP-OVA (p < 0.001) immunization (Figure 4C). These results indicate, in contrast to what has previously been suggested [48,49], that the memory CD8^+ ^T cell pool is maintained in the absence of type I IFN signalling.

In conclusion, TLR3 or MyD88-dependent innate signalling pathways are not crucial for the induction and activation of CD8^+ ^T cell responses after DREP and VREP immunization. However, IRF3, downstream of both TLR and RLR signalling pathways and important for the generation of type I IFNs and pro-inflammatory cytokines, was required for a potent T cell response after VREP immunization, with a similar trend in DREP immunized mice. In contrast, type I IFNs has a suppressing effect on the T cell response both after DREP and VREP immunization.

## Discussion

In this study we wanted to characterise what possible innate signals could form the basis of the enhanced immunogenicity of DREP and also to investigate if alphaviral replicons, delivered as DNA (DREP) or as viral particles (VREP), activate the same innate signalling pathways, a comparison that has not previously been done.

DREP vectors have been shown to induce stronger immune responses in comparison to convDNA vectors [32-37]. However, in those studies the different backbones in the vectors were not considered. In this study we constructed two new vectors, DREP-OVA and deltaREP-OVA, containing the same backbone, and compared their immunogenicity in mice. We confirmed that indeed DREP is more immunogenic than deltaREP on a per dose basis, despite the potential advantages for deltaREP, such as size. The size difference is in favour for the deltaREP construct, since a smaller plasmid size generates a higher transfection efficacy, as well as higher numbers of plasmid copies per μg in comparison to a bigger plasmid, such as DREP. Since the superior immune effects induced by DREPs does not depend on unusually high antigen expression levels ([1,2,32] and data not shown), it is probably mediated by a more potent activation of innate immune responses.

In this study we found that the CD8^+ ^T cell responses induced by DREP or VREP immunization were similar in wild type, TLR3 and MyD88 deficient mice. In accordance with this, it was recently shown that TLR3 is not crucial for the generation of a CD8^+ ^T cell response after DREP immunization [50] and this also confirms our earlier results that the T cell responses are not dependent on TLR3, nor MyD88, after VREP immunization [17,22]. In contrast, we have previously shown that the TLR3 signalling pathway is needed for VREP-infected cells to induce CD8^+ ^T cell responses *in vivo*. However, these results were obtained in a xenogenic model in which VREP infected Vero cells, lacking type I IFN production, were used for immunization [3]. Hence, our present results suggest that the TLR3 pathway is dispensable *in vivo *when type I IFN is present, induced by multiple signalling pathways after DREP and VREP immunization.

TLR3 signalling, as well as RLR signalling pathways, lead to activation of IRF3, which is known to play a critical role in antiviral responses [51,52] and crucial for induction of type I IFNs as well as pro-inflammatory cytokines. Interestingly, the CD8^+ ^T cell response was significantly lower in the absence of IRF3 after VREP immunization, with a similar trend detected after DREP immunization, albeit not statistically significant. These results indicate that replicon induced RNAs are important activators of innate signalling pathways and adaptive immune responses. In accordance, it was recently published that Chikungunya virus, an other alphavirus, activates IRF3 via interferon promoter stimulator 1 (IPS-1), a signalling molecule downstream of the RLRs [53]. It further indicates that multiple innate signalling pathways, compensating for each other, are activated after DREP and VREP immunizations, since the T cell response was not affected in single TLR KOs and/or that RLRs play a bigger role than TLRs in replicon induced immunity. In agreement, it was recently published that adenoviral vaccine vectors, which equally to alphaviral vaccine vectors induce strong immune responses, activate multiple innate signalling pathways [54]. Moreover, yellow fever vaccine 17D (YF-17D) is regarded as one of the most effective live attenuated vaccines available [55] and has been shown to activate several innate signalling pathways such as TLR2, 7, 8 and 9. Hence, it might very well be that activation of multiple innate signalling pathways is a feature of potent vaccines. The reason why we do detect a significant difference between IRF3KO and wild type mice after VREP-OVA immunization, merely detected as a similar trend after DREP-OVA immunization, is most probably due to differences in transfection/infection efficacy. DREP-OVA is mechanically forced into the muscle cells during injection whereas VREP-OVA is actively infecting the cells, most likely leading to a more effective and more reproducible antigen delivery into the cells by VREP-OVA, generally detected as less variation between the immunized mice.

The signalling of several TLRs (TLR3, 4, 7 and 9) and RLRs converge into a type I IFN response. Type I IFNs encompass a multitude of stimulatory effects on the adaptive T cell response including activation of DC function, promotion of cross-priming and stimulation of memory T cells [18-21]. We have previously shown that VREP is a potent inducer of type I IFN [22]. In the present study we observed that the CD8^+ ^T cell response was stronger in mice lacking a functional type I IFN system, and that this balance was maintained in the memory response. These results indicate that type I IFNs suppress the immune response and that the memory CD8^+ ^T cell pool is maintained in the absence of type I IFN signalling, in contrast to what has previously been suggested [18-21,48,49]. Moreover, it has previously been reported that splenocyte cultures from DREP immunized IFN-AR1 KO mice produce lower levels of IFNγ *in vitro*, in comparison to splenocytes from wild type mice [56]. However, our dose titration experiment showed that the T cell responses linearly increased with higher doses of DREP in the IFN-AR1 KO mice whereas, in the wild type mice, the immune responses did not increase at doses higher than 10 μg of DREP. This suggests that type I IFN is only stimulatory until a certain threshold level has been reached. This is in agreement with earlier findings that type I IFNs has a stimulatory effect on CD8^+ ^T cell responses at low doses, whereas at higher doses the cytotoxic response was suppressed [57]. The IRF3KO mice, in contrast to the IFN-AR1 KO mice, are defective in production of both type I IFNs and pro-inflammatory cytokines. Hence, the lower T cell response detected in the IRF3KO mice could be due to differences in other cytokines than type I IFNs, which compensate for the lack of type I IFNs in the IFN-AR1 KO mice.

The increased T cell response in the IFN-AR1 KO mice could have several explanations, such as abundant antigen production and/or the presence of more innate receptor ligands due to non-restricted RNA replication, as RNA viruses are prone to inhibition of replication by type I IFN. In agreement, plasmid DNA transgene expression has been shown to be inhibited by type I IFNs [58]. During a viral infection type I IFN induces an antiviral state in yet uninfected cells, thus prohibiting further spread of the infecting agent. However, in our case, replication of neither vector (VREP or DREP) results in production of new infectious particles. Thus, type I IFN mediated antigen or RNA replication suppression have to be in an autocrine fashion. However, Vero cells infected with VREPs and treated with type I IFN at different time-points post-infection expressed similar amounts of VREP encoded protein, indicating that type I IFN does not suppress the antigen expression level in the infected cell per se (data not shown). Moreover, we have previously shown that within a few hours after transfection, DREP and VREP replication will result in a type I IFN/PKR mediated shutdown of host protein synthesis, without affecting production of the vector encoded antigen *in vitro *[59-61]. In addition, we have also shown that replication of a VREP mutant, that induces high levels of type I IFNs, was not suppressed in comparison to wild type VREP *in vitro *[62]. Moreover, by increasing the DREP-OVA dose, antigen and innate receptor ligand load increase, but nevertheless the immune response does not increase in wild type mice immunized with doses exceeding 10 μg DREP-OVA (Figure 4B). Furthermore, it was recently published that addition of type I IFNs do not inhibit alphaviral replication once RNA replication has been established [63]. Collectively, these data suggest that type I IFN does not act in an autocrine fashion lowering replicon encoded antigen expression.

A role of type I IFNs is to activate negative feedback mechanisms to avoid prolonged cytokine production [64] and also to induce apoptosis [43,65]. These regulatory pathways are non-functional in IFN-AR1 KO mice and could explain the increased CD8^+ ^T cell responses in the absence of the type I IFN system. It has previously been shown that replicon induced apoptosis increase uptake of apoptotic bodies and cross-priming by DCs [66] and by blocking apoptosis, mice were less protected against a subsequent tumour challenge [43,67]. However, it was recently reported that co-delivery of pro-apoptotic genes reduced the efficacy of DNA vaccines [68]. Hence, type I IFN induced apoptosis could give two effects, either stimulating formation of apoptotic vesicles, thereby stimulating cross-priming, or lowering the antigen level produced, due to premature cell death. If type I IFN induced apoptosis is of importance in our system, the latter scenario must be at play in the wild type mice, lowering the antigen level and hence the immune response.

Despite the incapability of the IFN-AR1 KO mice to respond to type I IFNs they still produce and respond to other cytokines induced after DREP and VREP immunization. Hence, the robust T cell responses established despite the lack of the adjuvant effect from type I IFN is probably due to other effector mechanisms in play in the IFN-AR1 KO mice, sufficient for the induction of an immune response, in combination with lack of the negative feedback loop mediated by the type I IFN. In accordance, it was recently published that TLR ligands both positively and negatively modulate the immune response after viral vector immunization [69].

In conclusion, DREP immunization results in robust T cell responses even after a single administration and are much stronger than those obtained by immunization with convDNA vaccines. We found that DREP induced T cell responses were quite similar to those induced by VREP, suggesting that both vaccine platforms use the same innate signalling pathways. Even though our results could not conclude a single TLR to be crucial for the adjuvant effect induced by SFV replicons, we could show that IRF3, a signalling molecule downstream of several RNA receptors, was needed for a fullminant T cell response in VREP immunized mice, with a similar trend in DREP immunized mice. Our data suggest that alphaviral replicon-based vectors activate multiple innate signalling pathways contributing to their potent immunogenicity. Moreover, we show that VREP and DREP induced type I IFN restricts both primary and memory CD8^+ ^T cell responses. It would seem that the efficacy of the DREP and VREP vaccines, being RNA replicons sensitive to host cellular responses, are dependent on a balance between stimulatory and inhibitory signals where replicon induced RNAs and type I IFN play an important role.

## Competing interests

The authors declare that they have no competing interests.

## Authors' contributions

TN carried out all the experiments including designing the experiments, acquisition of data, analysis and interpretation of data. TN also drafted the manuscript. LK has helped with acquisition of data and some analysis of data. EN has helped with acquisition of some data and revising the manuscript. MC has helped with design of some experiments and revising the manuscript. PL has helped with design of some experiments, revised the manuscript and given final approval of the version to be published. All authors have read and approved the final manuscript.
